# Anatomy of a Transcatheter Mitral Valve Service

**DOI:** 10.3389/fcvm.2022.862471

**Published:** 2022-04-15

**Authors:** Harminder Gill, Heath S. L. Adams, Omar Chehab, Christopher Allen, Jane Hancock, Pablo Lamata, Gianluca Lucchese, Bernard Prendergast, Simon Redwood, Tiffany Patterson, Ronak Rajani

**Affiliations:** ^1^Cardiovascuar Directorate, Guy's and St Thomas' Hospitals, London, United Kingdom; ^2^School of Biomedical Engineering and Imaging Sciences Engineering, King's College London, London, United Kingdom

**Keywords:** transcatheter mitral valve replacement, mitral regurgitation, Heart Team, mitral edge-to-edge repair, indirect annuloplasty, mitral stenosis

## Abstract

Transcatheter mitral therapies offer treatment options to selected patients who are unable to undergo open procedures due to prohibitive surgical risk. Data detailing the design and structure of transcatheter mitral services to ensure appropriate patient selection and tailored management strategies is lacking. We report our initial experience of developing and running a purpose-built transcatheter mitral service. The nature and number of referral sources, the multi-disciplinary make-up of the dedicated Mitral Heart Team and the use of integrative imaging assessment with incorporation of computational solutions are discussed. In addition, a summary of the clinical decision-making process is presented. This report sets out a framework from which future clinics can evolve to improve and streamline the delivery of transcatheter mitral therapies.

## Introduction

The application of the Heart Team to facilitate transcatheter aortic valve replacement (TAVR) in patients with aortic stenosis is now established in routine clinical practice with well-defined patient pathways (1). It is appealing to consider that the application of this model to patients' inoperable mitral valve (MV) disease would be equally efficacious. Although up to 10% of patients above the age of 75 years have significant MR, only 15% undergo surgical treatment (2). This suggests that a substantial group of patients may be eligible for transcatheter mitral valve therapies (TMVT). Despite this, the number of patients being referred for TMVT is small and screening failure rates remain high, suggesting that alternative strategies are needed to best identify and treat these patients. In the following report we detail our experience in delivering a dedicated Transcatheter Mitral Valve (TMV) Service consisting of a dedicated TMV clinic alongside a specialist multi-disciplinary Heart Team meeting.

## Structure of the TMV Clinic

**Figure 1:**
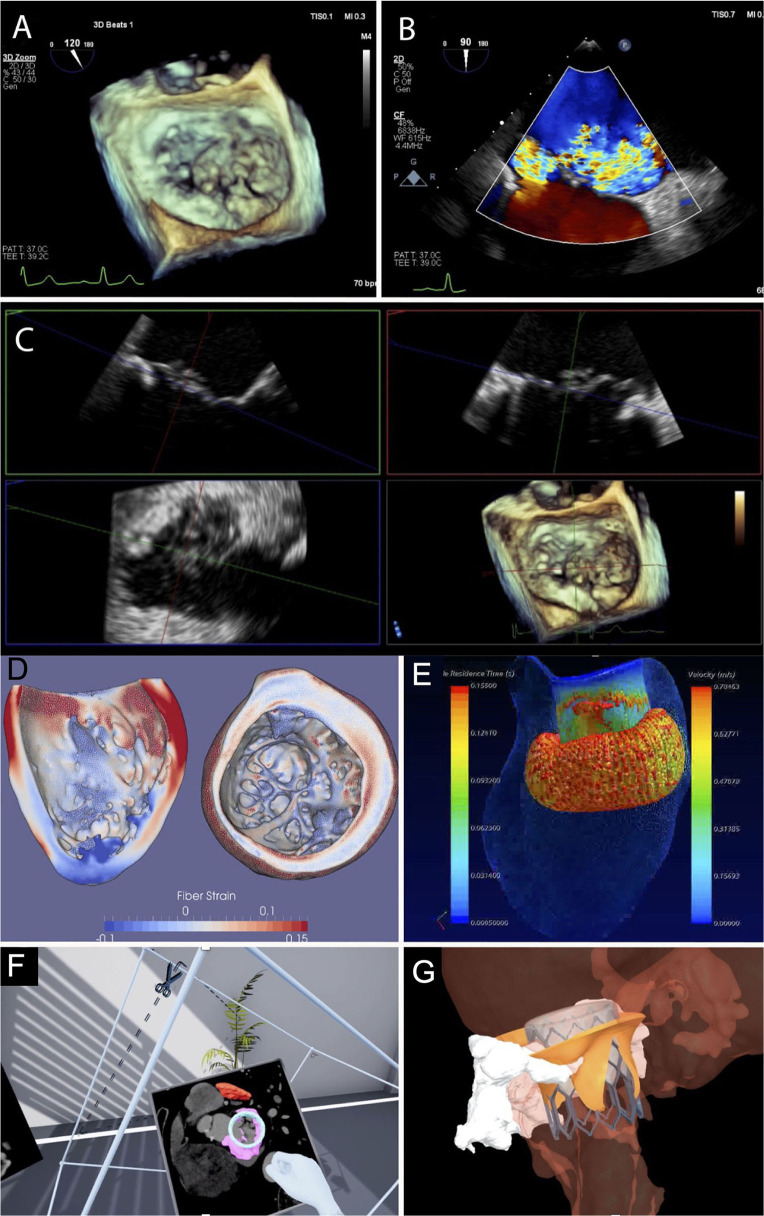
Transoesophageal echocardiogram for edge-edge mitral valve leaflet repair. **(A)** Shows 3D imaging, **(B)** Demonstrates Doppler and **(C)** Dual plane imaging. Advanced imaging processing techniques used within the TMVC. **(D)** Shows myocardial fiber strain from CT. **(E)** Demonstrates 4D CT flow to predict blood residence time within the left ventricle following TMVR device deployment. **(F)** Is an example of virtual reality. **(G)** Demonstrates virtual implantation and modeling. 3D, 3-dimensional; TMVC, transcatheter mitral valve clinic; CT, computed tomography; TMVR, transcatheter mitral valve replacement.

A one-stop TMV clinic was run by five Cardiologists. The first two (RR and JH) were imaging cardiologists (echocardiography and cardiac CT) with experience in managing patients with complex valve disease and heart failure, and the third, fourth and fifth were structural interventional cardiologists (TP, BP, and SR) with experience in transcatheter aortic and mitral therapies. Discussion at a Mitral Heart Team meeting comprising of a heart failure/imaging specialist, cardiac surgeon and interventional cardiologist was undertaken for all patients entering the TMT pathway. If treatment was indicated, but surgical options were deemed high risk, then assessment in the TMV clinic was organized. For other patients a clinic review was arranged to ensure transcatheter options were explored prior to resorting to medical therapy alone or palliation. Following approval for review, patients were seen in a purpose-designed, specialist TMV clinic. Integral to the clinic was the availability of a spectrum of TMVT devices. These comprised (1) transcatheter mitral valve replacement (TMVR) with an Intrepid (Medtronic, MN, USA) or Sapien-3 (Edwards Lifesciences, CA, USA) for cases valve in mitral-annular-calcification (VIMAC), valve-in-valve (VIV) or valve-in-ring (VIR), (2) transcatheter edge-edge repair (TEER) with MitraClip (Abbott, IL, USA) or Pascal (Edwards Lifesciences, CA, USA) for primary or secondary MR, and (3) indirect annuloplasty (IA) with an ARTO (MVRx, CA, USA) device. The clinic was implemented as a one-stop service where patients underwent clinical assessment, a 12-lead electrocardiogram, blood tests and echocardiogram. At this stage, one of the following recommendations was made: (A) no treatment required, (B) optimisation of medical therapy and referral for assessment by the heart failure specialist team, (C) device therapy, (D) re-discussion at the mitral Heart team meeting, or (E) for transcatheter mitral treatment if the prior conditions had been met. If at this stage the preferred strategy was felt to be TEER (i.e., primary or secondary mitral regurgitation with favorable anatomy for TEER), a same day transoesophageal echocardiogram was performed (Figure 1). For all other patients (VIMAC, VIV or VIR TMVR and IA), a multiphase ECG-gated CT and transoesophageal echocardiogram were planned for a subsequent visit. Following completion of clinical and imaging assessment, patients were discussed at a dedicated structural Heart Team meeting where patients were either approved for treatment or referred for further advanced imaging. Advanced processing included (A) collaboration with academic partners (King's College London) for cardiac CT 4D flow simulation to predict left ventricular outflow tract gradients following TMVR, (B) finite element modeling (FEM; FEops, Ghent, Belgium) for VIMAC predictions, and (C) preparation of imaging for CT-fluroscopic or CT-echocardiographic fusion for the intended procedure (Figures 1D–G) (3, 4).

**Figure 2:**
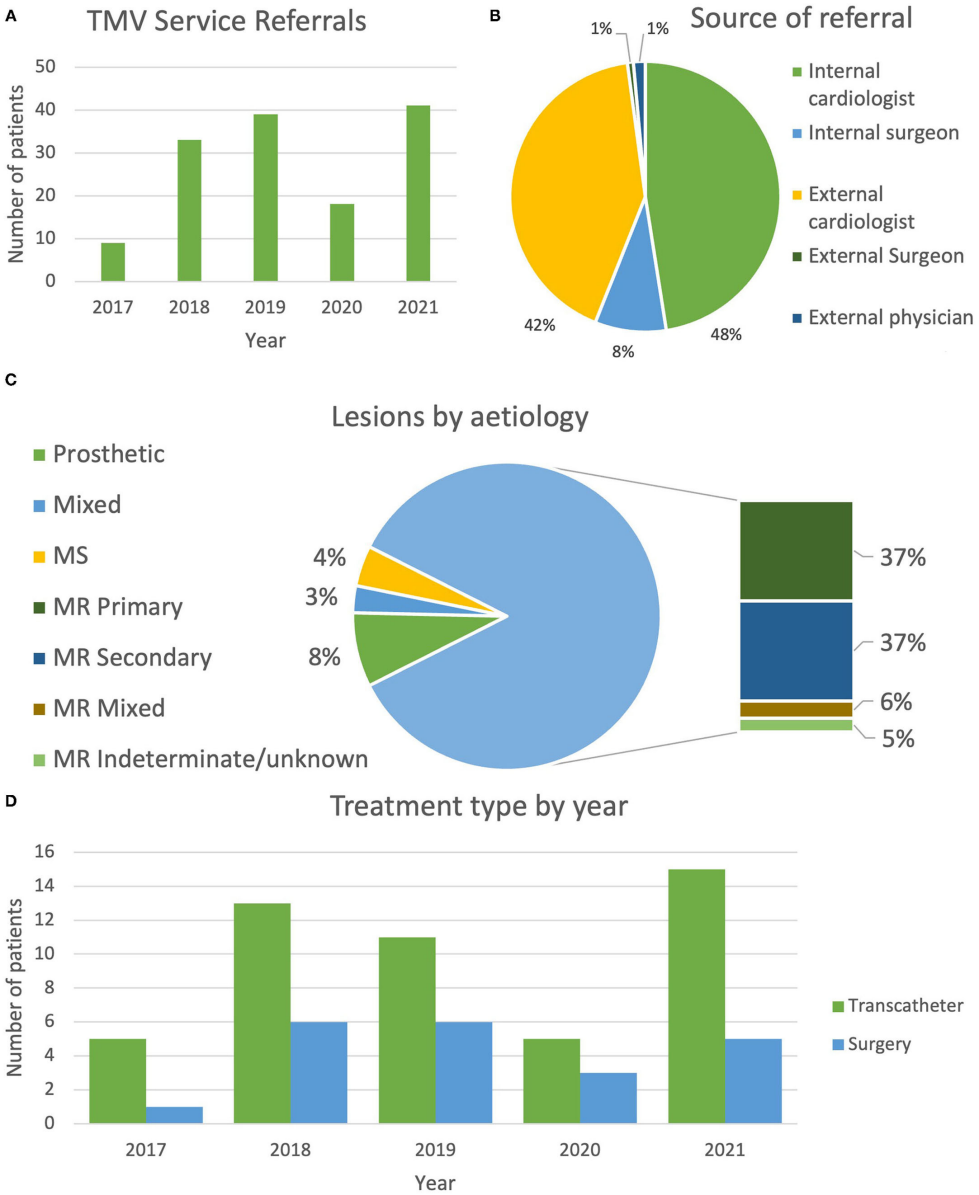
**(A)** Bar chart showing referrals to the TMVC by year. **(B)** Pie chart demonstrating referral sources to the transcatheter mitral clinic. **(C)** Pie chart demonstrating the mitral regurgitation as the dominant lesion assessed in the service, with equal numbers of primary and secondary mitral regurgitation referred. Prosthetic valve dysfunction, mitral stenosis or mixed mitral disease were less common. **(D)** Treatment type by year is shown. Here we can see a gradual increase in the transcatheter therapies offered. Surgery remains constant. 2020 saw a downturn in mitral procedures due to the COVID-19 pandemic. TMVC, transcatheter mitral valve clinic.

**Figure 3:**
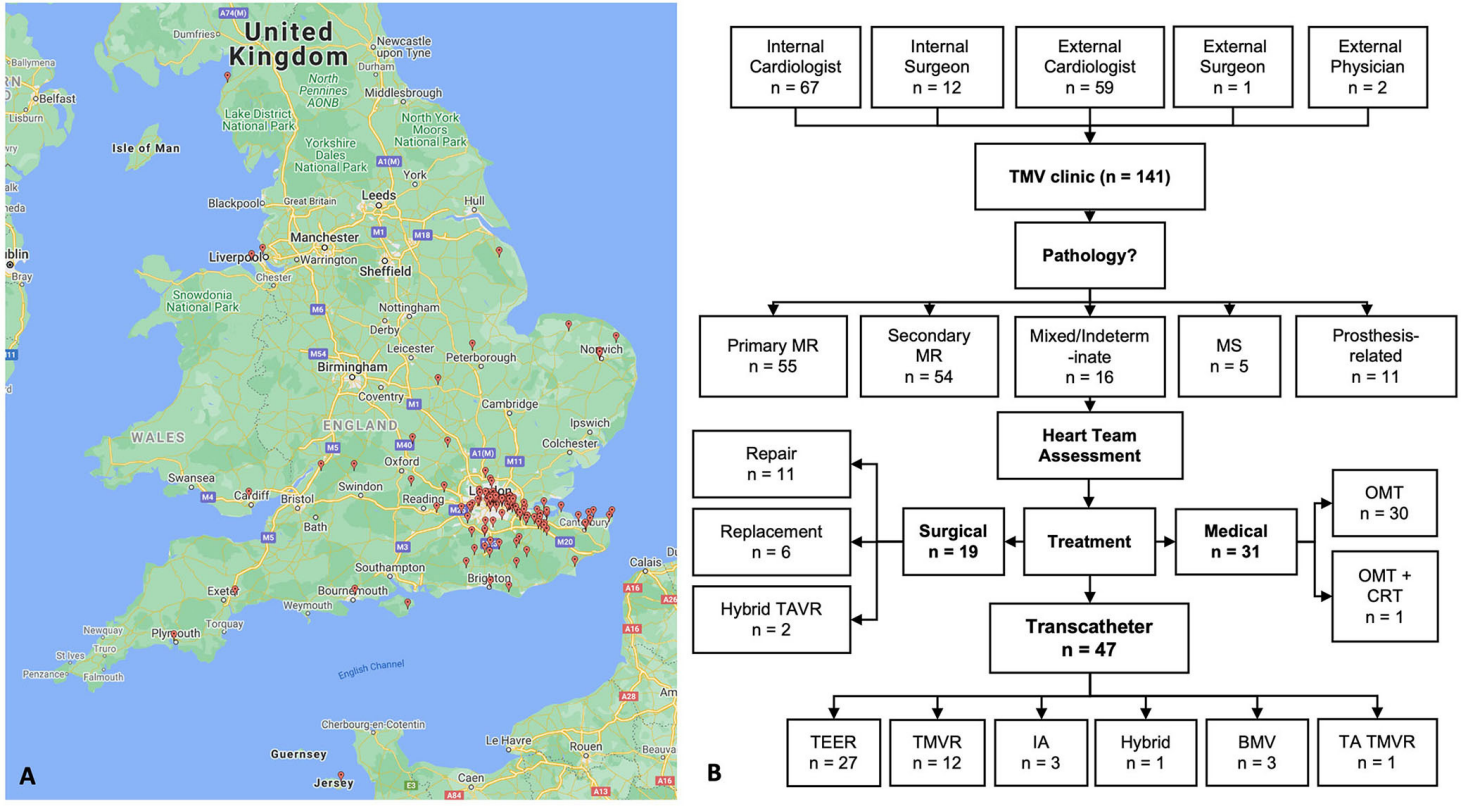
**(A)** Geographical referral sources that developed upon establishment of the TMV service in London. **(B)** Overview of the pathway from referral to assessment and treatment. TMV, transcatheter mitral valve; MR, mitral regurgitation; MS, mitral stenosis; TAVR, transcatheter aortic valve replacement; OMT, optimum medical therapy; CRT, cardiac resynchronisation therapy; TEER, transcatheter edge-edge repair; TMVR, transcatheter mitral valve replacement; IA, indirect annuloplasty; BMV, balloon mitral valvuloplasty; TA TMVR, transapical transcatheter mitral valve.

As awareness of the TMV clinic grew, there was a rapid increase in referrals which was only interrupted by the Coronavirus-19 pandemic (Figure 2A). The clinic reviewed 141 patients for TMVT from May 2017 to November 2021. Seventy-nine (56%) patients were referred internally by cardiologists or surgeons, whereas 42.5% were received from external clinicians (Figure 2B), including those from geographically distant locations (Figure 3A). There were two cases of direct referral by primary care physicians.

## Our Experience

Baseline characteristics are recorded in Table 1. The mean age was 77.5 years (range 46.9–95.3 years), of whom 74 (52.8%) were men. NYHA class III-IV symptoms were present in 125 (88.6%) of patients. Etiologies of mitral regurgitation are shown in Figure 2C. There was substantial mortality within the cohort and 8 (6%) patients died whilst awaiting assessment or treatment. Procedural management was delivered to a total of 66 (46.8%) patients. Surgical repair was the preferred strategy in 19 patients (13.4%), with repair in 11 patients, and replacement in 8. Transcatheter management was delivered in the remaining 47 patients: 27 patients underwent TEER (19.1%), 13 TMVR (9.2%), 3 IA (2.1%) and 1 Hybrid procedure (TEER + IA) (0.07%). Successful procedural outcomes were recorded in 44 patients (93.6%), with failure of 2 TEER and 1 IA procedures. Additionally, 3 patients suffering with MS were treated with balloon mitral valvuloplasty (BMV). In total, 18 (13%) patients were declined procedural treatment owing to minimal symptoms or scope for further medical optimisation. In 18 (12.7%) patients who met clinical criteria for treatment, there were no surgical or TMVT options available following appropriate imaging assessment (prohibitive surgical risk, small LV cavity, risk of LVOT obstruction, degree of MAC). A multitude of reasons prevented patients receiving definitive management and these are summarized in Table 2. An overview of the pathway is summarized in Figure 3B.

**Table 1:** Patient baseline characteristics.

		**TEER**	**TMVR**	**Surgery**	**OMT**
	*n*	26	13	19	31
Demographic	Age	80.1	76	72.1	76.1
	Male	18	8	12	16
	Female	9	5	7	15
Etiology	Primary MR	10	0	9	15
	Secondary MR	12	1	8	12
	Mixed MR	5	0	2	2
	Prosthetic	0	10	0	0
	MS	0	2	0	1
Echocardiography	LVIDD (mm)	56.0	53.2	54.2	53.2
	LVEF (%)	50.3	48.0	53.2	51.9
	Significant TR (%)	64	40	59	43
	Estimated PASP (mmhg)	51.3	50.8	51.1	53.9
Comorbidity	Hypertension (%)	38.5	25.0	47.0	43.4
	Coronary artery disease (%)	36.0	38.5	35.3	60.0
	Atrial fibrillation (%)	51.9	66.7	47.4	63.3
	Diabetes (%)	4.0	16.7	10.5	6.7
	CVA/TIA (%)	27.0	8.3	5.3	6.7
	Dialysis (%)	0.0	3.8	5.3	0.0
	Hb (g/dl)	118.3	123.2	123.5	126.0
	eGFR (ml/min)	51.8	64.0	71.2	51.0

*TTE, transthoracic echocardiogram; TEER, transcatheter edge-edge repair; TMVR, transcatheter mitral valve replacement; MR, mitral regurgitation; MS, mitral stenosis; LVIDD, left ventricular internal diameter end diastole; TR, tricuspid regurgitation (significant defined as moderate or greater); PASP, pulmonary artery systolic pressure; CVA, cerebrovascular accident; TIA, transient ischaemic attack; Hb, hemoglobin; eGFR, estimated glomerular filtration rate; OMT, optimum medical therapy*.

**Table 2:** Reasons for not receiving procedural management.

**Reasons for not receiving procedural management**	**Number of patients**
Treatment awaited pending clinician decision	4
Treatment awaited pending patient decision	1
Medical optimization and reassessment required	2
Clinic assessment or investigations awaited	3
Treatment awaited	6
Patient died before assessment	4
Patient died before treatment	4
No procedural treatment options after Heart Team Assessment	18
No indication for treatment	16
Treatment transferred to alternative center	4
Patient did not attend for appointment	4
Patient declined the treatment offered	9

## Discussion

There are several important observations from our experience in establishing a dedicated TMV service. The proportion of patients receiving comprehensive TMVT assessment increased from 9 in 2017 to 41 in 2021, despite a significant downturn in 2020 and early 2021 due to the COVID-19 pandemic, signaling a growing awareness and demand for the service. Moreover, participation in the clinic resulted in definitive procedural management for 46.8% of these patients. A modest increase in transcatheter therapy was noted, reflecting similar (but amplified) trends from US registry data (5). Our data demonstrate that the delivery of transcatheter therapies outstripped surgery (Figure 2D), which is unsurprising considering the cohort was already selected to favor TMVT by virtue of referral to the service.

The structure of our TMVs model was strategically designed to streamline the clinical decision-making process. Patients were often referred once symptomatic with substantial baseline comorbidity and frailty equating to poor physiological reserve. In such cases, rapid assessment and treatment is vital. To achieve this, we positioned a mix of specialists in the clinic as an entry point to the pathway with same day diagnostics for the majority of patients. We believe this bestowed greater referrer confidence in the clinic and increased referrals to the service. The incorporation of MV surgeons earlier in the pathway increased surgical confidence in the TMV clinic, enabling bilateral referral pathways with MV surgeons operating on patients initially deemed to be at high surgical risk, and patients who were not ideal for surgery being referred to the TMV clinic. The positioning of an imaging specialist at the fulcrum of the pathway enabled faster decision making and facilitated access to bioengineering solutions for pre-procedural planning and modeling.

**Figure 4:**
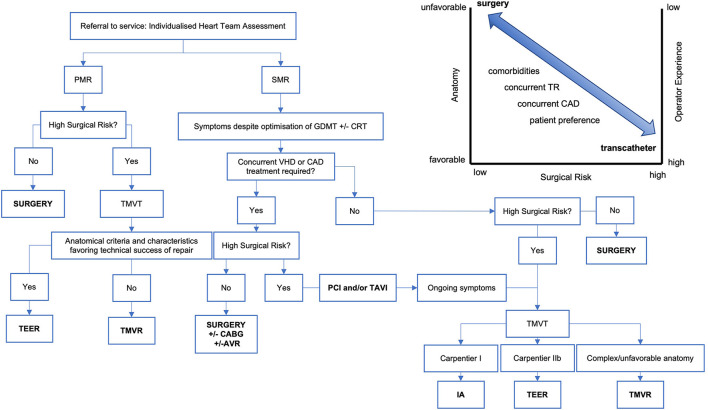
A simplified flowchart demonstrating the processes underpinning the patient journey to therapy for mitral regurgitation. The inset graphic illustrates surgical and transcatheter treatment lying on a continuum and how this decision is influenced by surgical risk, anatomic suitability, and operator experience with additional consideration given to concurrent cardiovascular conditions and patient preference. TMVT, transcatheter mitral valve therapy; PMR, primary mitral regurgitation; SMR, secondary mitral regurgitation; VHD, valvular heart disease; TAVR, transcatheter aortic valve replacement; GDMT, guideline-directed medical therapy; CRT, cardiac resynchronisation therapy; TEER, transcatheter edge-edge repair; TMVR, transcatheter mitral valve replacement; IA, indirect annuloplasty; CABG, coronary artery bypass grafting; PCI, percutaneous coronary intervention; AVR, aortic valve replacement.

Management of MR is complex, and a multitude of factors are considered when deciding the optimum management strategy (Figure 4). Clinical decision-making requires collaboration within the Heart Team to integrate patient-related factors such as symptomatic status, lesion severity and concurrent cardiac disease, in the context of procedure-related factors such as procedural risk, operator experience and anatomic suitability.

The means to assessing patient symptomatic status and lesion severity are well-established, however interpreting these findings in the context of concurrent cardiac comorbidity is vital. MR (especially SMR) is frequently accompanied by concurrent significant tricuspid regurgitation (TR). In the COAPT cohort, greater than moderate TR conferred an additional mortality and morbidity risk (6). Therefore, management of concurrent TR is an important consideration for patients undergoing treatment for MR. Severe pulmonary hypertension, significant (>moderate) right ventricular (RV) dysfunction, or TR requiring operation were exclusion criteria for the COAPT trial and similarly patients within our pathway were not managed procedurally where severe pulmonary hypertension or significant RV dysfunction were present (6). Isolated treatment of MR results in reduction of TR, however whether this result is maintained long-term is unclear (7). Interestingly, Besler et al. reported improved haemodynamic (LV and RV stroke volume and cardiac index), biochemical (reduction in N-terminal pro-brain natriuretic peptide) and symptomatic (NYHA class, 6 min walk test) indices after combined mitral and tricuspid TEER compared to isolated mitral TEER, with no significant difference in mortality (8). Retrospective analysis of patients from the TriValve and TRAMI registries suggested a mortality benefit at 1-year of a combined procedure rather than isolated MR or TR treatment (9). Further work is required to clarify the importance of managing TR in the context of MR in larger scale, randomized trials.

Treatment of concurrent coronary artery disease (CAD) is another important factor in the management of MR, and particularly relevant in ischaemic MR, and warrants address prior to definitive valve treatment (10). Within our pathway, if revascularisation was indicated but surgical risk was prohibitive, percutaneous coronary intervention was undertaken, with subsequent reassessment of the patient clinical status, symptoms and severity of MR. Alternatively, individuals with acceptable surgical risk, would be offered combined CABG and surgical MV where appropriate.

Whilst primary mitral regurgitation (PMR) requires treatment of the valve by repair or replacement once thresholds are met, pharmacotherapy is a core component of secondary mitral regurgitation (SMR) management. Within our cohort, 30% patients received optimum medical therapy, with one patient additionally receiving cardiac resynchronisation therapy (CRT). Optimisation of medical therapy yields improvements in LV function, severity of mitral regurgitation and LV geometry (11). This has a notable benefit in mortality and remains a bastion of SMR management. However, the shortfall in achieving target doses of prognostic medications in the undifferentiated heart failure population has been well documented. For instance, in the CHAMP-HF registry, where the key inclusion criteria was an EF <40%, target doses were achieved in 18.7, 10.8, and 2% of the cohort for beta blockers, angiotensin converting enzyme inhibitors/angiotensin receptor blockers and anti-neprolysin inhibitors, respectively, despite there being no contraindication or hypotension to prevent administration (12). This shows that barriers remain in achieving optimum medical therapy despite clear target doses, and comorbidity and patient tolerance are important limiting factors. Similarly, the impact of CRT in SMR to restore both local and global coordinated contraction has been reported to increase coaptation forces, reduce tethering forces, improve annular geometry and reduce diastolic MR (13). Whilst resulting in a modest reduction in MR, an improvement confers a mortality benefit, whereas failure to respond is associated with poorer outcomes (11).

Treatment strategy is dependent upon the accurate delineation of anatomic suitability. Careful assessment using transthoracic and/or transoesophageal echocardiography is indicated. Aside from important but generic information on the LV/RV volumes and function, valuable information impacting feasibility for TMVT is obtained. This includes coaptation height, tenting area, leaflet tethering, calcification, posterior leaflet height, annulus geometry and valve area. Important anatomical criteria along with cautions for different TMVT are included in Table 3.

**Table 3:** Indication and cautions for transcatheter mitral therapies.

	**Transcatheter edge-edge repair**	**Indirect annuloplasty**	**Transcatheter mitral valve replacement**
Devices	Mitraclip™ (Abbot) Pascal™ (Edwards)	Arto™ (MVRx)	Sapien 3™ (Edwards) Intrepid™ (Medtronic)
Indications	PMR, SMR	SMR	PMR, SMR
Anatomic considerations	MVA > 3 cm^2^ Central A2/P2 No calcification Grasping length > 10 mm Tenting Height <10 mm For a flail segment: flail width <15 mm, flail gap <10 mm, LVESD > 55 mm* For tethering: coaptation length <11 mm, overlap length > 2 mm	Annular Dilatation Predetermined by the core lab/PI Coronary sinus proximity and coplanarity	MVA 1.0–3.0 cm^2^ Multi-segment disease Commissural Disease Perforations Clefts Valve-in-ring Valve-in-valve Valve-in-MAC
Functional considerations	Mean gradient <4 mmHg		LVEF ≥ 30% Mean gradient 5–10 mmHg Low risk for LVOT obstruction
Cautions and contraindications	Leaflet perforation or clefts Severe calcification of the annulus or leaflets Barlow/rheumatic valve Flail width > 15 mm Flail gap >10 mm LVESD > 55 mm in PMR, or greater > 70 mm in SMR MS MVA <3.0 cm^2^ Mean gradient > 5 mmHg	Severe annular calcification	Access constraints Cardiomyopathy LVEDD > 70 mmHg Severe MS Fused commissures Severe MAC Bleeding/coagulation disorders RV dysfunction Severe LV dysfunction Significant CAD

*PMR, primary mitral regurgitation; SMR, secondary mitral regurgitation; MS, mitral stenosis; LVOT, left ventricular; MAC, mitral annular calcification; MVA, mitral valve area; RV, right ventricle; LV, left ventricle; CAD, coronary artery disease; PI, principal investigator; LVESD, left ventricular end-systolic diameter, outflow tract; LVEDD, left ventricular end-diastolic diameter*.

Assessment of surgical risk presented an additional challenge when administering the TMV service. Within our institution, surgical risk was estimated by Heart Team consensus, as suggested in ESC guidance (10). In our team's experience, the STS and Euroscore overestimate risk and serve as deterrent to offering procedural management; contributing to the widely reported undertreatment of MR. There is sparse data to underpin the best approach to assessing overall operative risk specific to transcatheter procedures, with surgical scores tending to overlook frailty of patients, and missing important features that impact transcatheter procedures (e.g., access, etc.). For example, Compagnogne et al. showed that Log Euroscore, Euroscore II and STS overestimated risk and were unable to stratify 30-day mortality in patients undergoing TAVR (14). German registry data suggests that the performance of conventional surgical risk scores is mediocre for patients undergoing TAVR with a tendency to overestimate risk, but even specialized transcatheter risk scores demonstrated only moderate performance at predicting 30-day mortality (15). Although this offers some insight into the limitations of such scores, both the pathogenesis of aortic stenosis and the procedural techniques differ and therefore the findings cannot be extrapolated unreservedly. The Mitral Regurgitation International Database (MIDA) score developed for risk stratification in degenerative mitral regurgitation successfully predicted 2-year all-cause mortality and heart-failure hospitalization in patients undergoing TEER in a multicentre, observational study, irrespective of mitral regurgitation etiology, with hazard of all-cause mortality increasing by 13% (95% CI 3–25%) for each additional point on the 12 point scale (16). Further work is required in prospective studies to assess the utility of this score. Ultimately, this highlights the importance of individualized risk assessment, provided by careful evaluation by experienced members of the Heart Team. Evaluation of current and novel risk stratifying methods for TMVT remains an area to be explored.

There is mounting evidence that operator experience and institutional case load influences the technical success and clinical outcomes of TMVT (17). For instance, analysis of 24,709 patients undergoing TEER in the German **TRA**nscatheter **MI**tral (TRAMI) registry, revealed that centers performing TEER in <25 patients/year had comparable in-hospital mortality to those performing >25 procedures/year and that this was the case with cut-offs at 10 and 50 procedures a year (18). However, analysis over the entire 7-year period showed that centers performing <300 TEER, had a range of in-hospital mortality of 0–20%, whereas in those undertaking >300 TEER, the in-hospital mortality ranged 0.9–5.5% (18). Evaluation of the US TVT registry demonstrated a higher likelihood of procedural success, reduced procedure times and fewer complications with increasing institutional case experience (19). This highlights the importance of achieving the requisite volume and suggests that TMVT is best administered in larger, centralized cardiac centers.

Novel approaches to Heart Team dynamics may enable greater access to treatment and improved outcomes for patients with MV disease being considered for TMVT. Further evaluation of TMVC models is needed to confirm this hypothesis.

## Data Availability Statement

The datasets presented in this article are not readily available since they would permit patient identification. Requests to access the datasets should be directed to ronak.rajani@gstt.nhs.uk.

## Ethics Statement

Ethical review and approval was not required for the study on human participants in accordance with the local legislation and institutional requirements. Written informed consent for participation was not required for this study in accordance with the national legislation and the institutional requirements.

## Author Contributions

HG, HA, BP, and RR: concept, design, and drafting. HG and RR: database and analysis. OC, CA, JH, PL, GL, BP, SR, and TP: critical review, editing, and revising manuscript. All authors contributed to the article and approved the submitted version.

## Funding

This study was supported by BHF Translational Award Ref: TG/17/3/33406; EU's Horizon 2020 R&I program under the Marie Skłodowska-Curie g.a. No. 764738; Wellcome Trust/EPSRC Centre for Medical Engineering (WT 203148/Z/16/Z). PL holds a Wellcome Trust Senior Research Fellowship (209450/Z/17/Z).

## Conflict of Interest

The authors declare that the research was conducted in the absence of any commercial or financial relationships that could be construed as a potential conflict of interest.

## Publisher's Note

All claims expressed in this article are solely those of the authors and do not necessarily represent those of their affiliated organizations, or those of the publisher, the editors and the reviewers. Any product that may be evaluated in this article, or claim that may be made by its manufacturer, is not guaranteed or endorsed by the publisher.
